# A Brief Online Program Integrating Mindfulness and Stretching Exercises: Effects on Well‐Being in Health Sciences Students

**DOI:** 10.1002/jclp.70034

**Published:** 2025-08-13

**Authors:** Zeynep Ayça Terzioğlu, S. Gülfem Çakır‐Çelebi

**Affiliations:** ^1^ Department of Pilotage, School of Civil Aviation Antalya Bilim University Antalya Türkiye; ^2^ Department of Guidance and Counselling, Faculty of Education Akdeniz University Antalya Türkiye

**Keywords:** exercise psychology, health sciences students, mental well‐being, mindfulness, self‐compassion

## Abstract

**Objective:**

Students enrolled in health sciences programs, similar to practicing healthcare professionals, are at high risk for burnout. Despite the numerous challenges these students face, it is crucial to support their well‐being. This study examined the impact of a brief online intervention that combined mindfulness and stretching exercises on mindfulness, self‐compassion, and mental well‐being among health sciences students.

**Method:**

The study employed a randomized control group design with pre‐test, post‐test, and follow‐up assessments. The study group consisted of 38 undergraduate students in the health sciences. The participants in the experimental group attended a six‐session online program that integrated mindfulness and stretching exercises. In contrast, the participants in the control group did not receive any treatment. Data were collected using the Five Facet Mindfulness Questionnaire‐Short Form, Self‐Compassion Scale‐Short Form, and Warwick‐Edinburgh Mental Well‐Being Scale.

**Results:**

The findings revealed that following a six‐session online program combining mindfulness and stretching exercises, there were significant differences in mindfulness, self‐compassion, and mental well‐being scores between experimental and control groups over time. The experimental group showed statistically significant improvements in mindfulness, self‐compassion, and mental well‐being compared with the control group.

**Conclusions:**

These results indicate that a brief mindfulness‐based stretching exercises program delivered online can effectively enhance mindfulness, self‐compassion, and mental well‐being among undergraduate students pursuing health sciences. The results support the potential of such interventions to promote psychological well‐being among health sciences students.

**Clinical trial registration:**

This study was not pre‐registered.

## 1 Introduction

College years represent a time when students face the responsibilities of making choices and adopting lifestyle habits while also encountering the difficulties associated with emerging adulthood. University students may face the stress of achieving their academic goals, which increases demands and burdens and leads to further stress (Pozos‐Radillo et al. 2014). Throughout this period, college students must deal with the challenges of managing their mental health, educational goals, and relationships with others. These circumstances can have a substantial impact on university students' overall well‐being. Students enrolled in higher education, particularly those pursuing health‐related degrees, appear to have more demanding academic experiences, resulting in diminished overall well‐being (Bagcivan et al. 2015; Franzen et al. 2021; Tran et al. 2022; Zhao et al. 2015). An examination of the existing research indicates that multiple factors contribute to explaining psychological well‐being, with online interventions serving as crucial tools for promoting well‐being (Krifa et al. 2022). Intensive academic programs (Divaris et al. 2008) professional expectations (Batyrbekova et al. 2022; Diderichsen et al. 2011), and personality traits (Henning et al. 1998) can create various physical and psychological challenges for health sciences students during their education. In this context, there is growing interest in mindfulness practices to enhance students' well‐being (Braun et al. 2020; Ross et al. 2020).

Research has shown that mindfulness is positively related to self‐compassion, and this relationship plays a mediating role in an individual's psychological resilience (Baer et al. 2006; Neff and Germer 2013). Self‐compassion refers to the development of a kind and understanding attitude towards oneself when faced with failure or challenging experiences (Neff 2003). This compassionate attitude contributes to reducing stress, anxiety, and depression by reducing internal criticism. Mindfulness practices strengthen an individual's capacity to cope with negative emotions by increasing self‐compassion and supporting mental well‐being at the end of this process (Kuyken et al. 2010). The finding that self‐compassion partially mediates the relationship between mindfulness and mental well‐being emphasizes the importance of this mechanism (Hollis‐Walker and Colosimo 2011; Zessin et al. 2015). In a study conducted by Zollars et al. (2019), a significant increase in mindfulness level and mental well‐being, and a decrease in perceived stress level were observed in participants who performed mindfulness meditation. Cowand et al. (2024) found that self‐compassion can positively affect not only psychological well‐being but also physiological stress responses. In this case, self‐compassion may be an important protective factor for university students in coping with stress. The successful outcomes of mindfulness meditation, even during stressful times like exam weeks, indicate that mindfulness practices are a practical approach to enhancing students' psychological resilience in demanding academic settings. Further,

In addition to mindfulness and self‐compassion, engaging in physical exercise has been recognized for its supportive role in improving mental health. A systematic meta‐analysis study has shown that chronic stress and unhealthy lifestyles can increase the risk of many diseases by disrupting the autonomic nervous system balance, but exercise, when administered at the appropriate dose and form, has regulatory and protective effects on the autonomic nervous and immune systems (Daniela et al. 2022). Stretching exercises have been found to increase vagal tone in the postexercise period, which may positively affect heart health and stress regulation (Farinatti et al. 2011). It has been shown that the positive effects of self‐compassion in the context of physical activity are more pronounced, especially in more intense types of exercise (Zhang et al. 2023). Exercise may not only reduce the risk of cardiovascular disease but also potentially improve the long‐term outcomes of mood disorders (Hearing et al. 2016). Individuals with high trait mindfulness have increased positive well‐being and experience less psychological distress during exercise, making them more inclined to engage in physical activity (Zhao and Gan 2025).

Research has demonstrated that individual characteristics such as mindfulness (Whitehead et al. 2019) and self‐compassion (Hall et al. 2013) play a significant role in elucidating psychological well‐being. Research spanning 6 years revealed that university students who underwent mindfulness‐based training experienced improved levels of subjective well‐being (De Vibe et al. 2018). In a related study, researchers discovered that among college students, those with higher self‐compassion reported greater well‐being and reduced distress (Fong and Loi 2016). In this context, mindfulness‐based practices can be considered a sustainable and effective psychological resilience tool, especially for students under intense academic pressure, as they support mental well‐being by developing self‐compassion and increasing their ability to cope with stress. Furthermore, it is evident that physical exercise is another important factor in enhancing psychological well‐being among university students (Herbert et al. 2020). Therefore, it is thought that combining mindfulness and self‐compassion‐based approaches with physical exercise will help individuals achieve holistic well‐being at both the psychological and physiological levels. In this study, we examined the impact of a 6‐week online program combining mindfulness and stretching exercises on health sciences students. Our investigation focused on assessing the changes in participants' mindfulness, self‐compassion, and overall mental well‐being as a result of this intervention.

### 1.1 Mindfulness, Self‐Compassion and Physical Exercise

Mindfulness involves being fully present in the present moment while avoiding being sucked into one's thoughts, feelings, or preferences (Kabat‐Zinn 2001, 2013). Mindfulness entails intentionally sustaining awareness of one's present‐moment encounters with an open mindset, aiming to foster utmost happiness for both oneself and others (Muelrath and Burk 2021). Research has shown that engaging in mindfulness practices can enhance psychological well‐being (Terzioğlu et al. 2024; Van Gordon et al. 2014). Recently, interventions based on mindfulness have gained popularity as a beneficial approach for enhancing mental health (Wielgosz et al. 2019) and fostering self‐compassion (Ondrejková et al. 2022).

Self‐compassion involves wanting health and well‐being for oneself, and acting proactively to improve one's situation. This implies that individuals think that their own problems are important and worthy of attention (Neff 2011). In addition, self‐compassion is about being kind to oneself when one has a difficult time or when something makes one feel bad (Bluth 2020). Research has shown that students in medicine, dentistry, and pharmacy who practiced mindfulness techniques experienced notable improvements in managing stress related to studying and exams, as well as in enhancing their ability to cope effectively. A self‐care program for health professionals resulted in students experiencing a shift in their perceived ability to enhance self‐care practices and a decrease in stress levels associated with studying and exams (Ross et al. 2020). Rakel and Rakel (2016) stated that preventive mental health has three main pillars. The first is what the individual adds to one's body, such as nutrition, medications, and supplements; the second is how the individual moves one's body, such as exercise and manual therapies; and the third is how the individual perceives the world, that is, the mind‐body connection.

Physical exercise is another key element for promoting a healthy lifestyle. Researchers have explored and documented a strong link between engaging in physical activity and improving overall well‐being (Bernstein and McNally 2017; Edwards 2006; Herbert et al. 2020). For instance, Edwards (2006) demonstrated that engaging in regular physical activity is positively correlated with overall well‐being scores and various components of well‐being, including emotional state, coherence perception, resilience, stress management, and coping abilities. Another study indicated that exercise could mitigate depression and perceived stress, with regular physical activity showing negative correlations with psychosomatic stress, self‐reported anxiety, and depression, and positive correlations with quality of life and positive affect (Herbert et al. 2020). Physical activity has been demonstrated to enhance an individual's capacity for emotion regulation (Bernstein and McNally 2017) and to improve physical and psychological well‐being (Zayed et al. 2018). Although the human body constantly transmits sensory information, individuals may not always be consciously aware of this process. Mindfulness is thus viewed as a way to re‐establish a connection with one's body, enabling individuals to become more attuned to the signals their body is sending (Thackray 2020). Mind‐body therapies are comprehensive treatments founded on the concept that the mental and physical aspects of a person affect each other's health and overall well‐being. Mind‐body therapies include meditation, stress management, yoga, biofeedback, Tai Chi, and mindfulness (Phelps and Hassed 2011).

Mind‐body therapies are proposed to promote health and well‐being through the integration of top‐down and bottom‐up processes that facilitate bidirectional interactions between the brain and body. Yoga, which consists of meditation, breathing exercises and physical practices, enables physiological and emotional regulation through the central nervous system (Taylor et al. 2010). Polyvagal Theory helps to understand how these processes work in social interaction, trauma and therapy by explaining the automatic responses of the nervous system according to the perception of safety or threat. Behaviors are mostly determined not consciously but by physiological reactions based on the perception of bodily safety (Dana 2018; Porges 2015). Since mind‐body therapies affect vagal pathways, regular activation of these pathways through practices, such as breathing exercises, supports physiological, emotional, and social self‐regulation and resilience (Schmalzl et al. 2015; Porges 2017). Exercise balances cortisol levels by modulating the hypothalamic‐pituitary‐adrenal (HPA) axis, thus increasing stress tolerance and supporting vagal tone (Zschucke et al. 2015). It has been shown that a stretching protocol including multiple exercises and sets can increase vagal activity after exercise in individuals with low flexibility (Farinatti et al. 2011). This indicates that the body begins to recover after stretching and the autonomic nervous system returns to balance. That is, the increase in vagal activity after stretching exercise reflects the regulatory and calming effects of exercise. Similarly, in terms of polyvagal theory, Mindfulness‐Based Movement (MBM) provides physical and emotional balance through exercise and social interaction, and creates regulatory effects on the autonomic nervous system (Lucas et al. 2018).

Upon reviewing the literature, it is evident that the polyvagal theory offers a comprehensive theoretical framework for understanding the neurophysiological underpinnings of mindfulness‐based interventions and physical exercise, both of which contribute to enhancing individual well‐being. Polyvagal theory offers powerful theoretical explanations for the neurophysiological underpinnings of mindfulness‐based approaches and physical exercise that support individual well‐being. These practices both provide safety‐based reorganization of the autonomic nervous system and increase the capacity to cope with stress by strengthening self‐regulatory functions in the brain structures. Therefore, the integration of mindfulness‐based therapies and physical exercise in educational settings is thought to support students' psychological well‐being.

### 1.2 Health Sciences Students

The health sciences field encompasses both theoretical knowledge and practical applications. Education in this discipline involves a combination of conceptual learning and hands‐on experiences, reflecting its practice‐oriented nature. Students may encounter various difficulties in their education, especially in clinical settings (Humphris et al. 2002; Jonsén et al. 2013; Naidu et al. 2002). A study by Dalir and Mazloum (2012) on nursing and midwifery students revealed that approximately one‐third of them experienced poor mental health. The same study also reported that students' lack of enthusiasm for their chosen field of study negatively affected their mental well‐being. Problems experienced in the educational process at different levels not only affect the emotional and social domains of students, but can also create significant obstacles in the acquisition of professional skills.

Research has revealed that students in nursing and midwifery programs experience academic stress. The primary factors contributing to this stress included challenges in comprehending course material, an overloaded curriculum, extended lecture periods, and insufficient time for relaxation (Osei et al. 2022). In another study, burnout syndrome was found to increase among midwifery students as their career years progressed (López‐Alegría et al. 2020). In this case, health sciences students can be considered a population at high risk of burnout as much as health professionals. Despite the problems experienced at different levels, it is important for students to maintain their well‐being. Mhalkar et al. (2014) reported that nursing students who took part in a program designed to enhance emotional intelligence exhibited greater advancements in self‐efficacy (personal, interpersonal, and intrapersonal), overall emotional intelligence, and coping mechanisms. Following the completion of the program, the participants implemented more effective coping strategies, including positive reassessment and reaching out for social assistance.

A study examining the effects of mindfulness‐based stress reduction programs on nursing students demonstrated significant positive changes in their mental well‐being. The findings indicated that participants experienced substantial decreases in depression, anxiety, and stress levels along with an enhancement in mindfulness (Song and Lindquist 2015). Self‐compassion also prevents burnout among medical and dental students (Pereira et al. 2022). It is important for health sciences students to promote self‐help responses by enabling them to increase their compassion and awareness so that they can help their patients in the face of difficulties. Furthermore, developing compassion skills is crucial for students to handle and accept patients' suffering in clinical environments. Notably, as students experience increased psychological distress, they become less likely to seek assistance; however, they are inclined to utilize online well‐being support programs (Ryan et al. 2010). A comparable investigation revealed that Internet‐based positive psychology interventions significantly enhanced positive mood and reduced negative emotions among students, with beneficial effects persisting at both 3‐ and 6‐month follow‐up assessments (Liu et al. 2021). Consequently, this study examined the impact of an online program on students in health sciences.

### 1.3 Current Study

A previous study demonstrated that an eight‐session online mindfulness‐based physical exercise (MBPE) program successfully improved life satisfaction and psychological well‐being in university students during the COVID‐19 pandemic (Terzioğlu et al. 2024). This study aimed to evaluate the efficacy of a shortened and revised version of the MBPE program. Several studies have highlighted the importance of brief mindfulness interventions (Kang et al. 2023; Keng et al. 2015). For instance, Demarzo et al. (2017) examined the efficacy of short and long versions of a mindfulness‐based intervention (MBI) program and concluded that both formats outperformed the control group with comparable effect sizes. Significant improvements were observed in both forms compared with the control group at post‐tests and 6‐month follow‐ups, with no differences between the short and long forms. Research findings indicate that a month‐long mindfulness program substantially reduced symptoms of depression, anxiety, and general psychiatric distress among medical students (Keng et al. 2015). The intervention decreased the perceived stress levels and improved subjective happiness and life satisfaction. Berghoff et al. (2017) reported that both 10‐min and 20‐min mindfulness meditation interventions reduced stress and increased mindfulness over 2 weeks, with no significant difference between groups in total meditation days or time spent. However, the 20‐min group experienced greater increases in self‐compassion than the 10‐min group did. Based on prior research on short mindfulness interventions, a short version of the MBPE may yield positive outcomes in areas such as mindfulness, self‐compassion, and psychological well‐being. This study aimed to investigate the effects of a six‐session online program integrating mindfulness and stretching exercises on mindfulness, self‐compassion, and mental well‐being among health sciences students. The primary hypothesis posited that there would be a significant interaction between group and time, indicating that the experimental group would experience significantly greater enhancements in mindfulness, self‐compassion, and mental well‐being from pre‐test to post‐test compared to the control group. Furthermore, it was hypothesized that there would be no significant difference between participants' post‐test and follow‐up scores in the experimental group regarding mindfulness, self‐compassion, and mental well‐being.

## 2 Methods

The research design employed in this study was a randomized controlled experiment, featuring pre‐test, post‐test, and follow‐up assessments.

### 2.1 Participants

The research participants consisted of 38 university students who volunteered for the study and were from different health sciences departments (physiotherapy and rehabilitation, midwifery, nutrition, dietetics, and dentistry) at a university in southern Turkey. To be eligible for this study, participants had to meet the following requirements: (1) they were not regular practitioners of meditation, yoga, or physical exercise; (2) they had no prior experience attending a mindfulness‐based psychoeducation group; and (3) they volunteered to participate in the research. Data collection was conducted via e‐mail. The participants were 35 women and three men. The experimental group consisted of 18 females and one male, whereas the control group comprised 17 females and two males. Participants in both the experimental and control groups ranged in age from 18 to 22 years, with a mean age of 19.22 years.

### 2.2 Data Collection Instruments

#### 2.2.1 Five Facet Mindfulness Questionnaire ‐ Short Form

The Five Facet Mindfulness Questionnaire (*FFMQ‐S)* was originally developed as 39 items by Baer et al. (2006). Tran et al. (2013) shortened the scale from 39 items to 20 items. The short form adaptation study to Turkish culture was carried out by Ayalp and Hisli‐Şahin (2018). The scale consists of five sub‐dimensions. Cronbach's alpha values obtained for each subscale of the scale and for the total scale were 0.85, 0.76, 0.71, 0.69, 0.69, and 0.71, respectively.

#### 2.2.2 The Self‐Compassion Scale – Short Form

The Self‐Compassion Scale‐Short Form (Raes et al. 2011) is a shortened version of the Self‐Compassion Scale, developed by Neff (2003). Barutçu‐Yıldırım et al. (2023) adapted the scale to Turkish culture and examined its psychometric properties with a sample of university students. CFA was conducted to test the one‐factor structure of the scale, and the results showed that the model fit was adequate [(χ²/(48) = 117.778, *p* < 0.05, χ²/df = 2.45, CFI = 0.94, TLI = 0.91, RMSEA = 0.085, *p* < 0.05, SRMR = 0.05]. Cronbach's alpha coefficients for the total scale score were 0.86 and 0.90 for two different samples of university students, indicating a high internal consistency coefficient.

#### 2.2.3 Warwick‐Edinburgh Mental Well‐Being Scale

The Warwick‐Edinburgh Mental Well‐Being (WEMWBS) scale developed by Tennant et al. (2007) was adapted to Turkish culture by Keldal (2015). The scale consists of 14 positive items. CFA was conducted to test the one‐factor structure of the scale and the results showed that the model fit was adequate [(χ² = 271.55, sd = 73, χ²/sd = 3.71, NFI = 0.94, RFI = 0.93, IFI = 0.96, CFI = 0.96, NNFI = 0.95, RMR = 0.054]. The Cronbach's alpha for the scale was 0.92.

#### 2.2.4 The Program Evaluation Form

The researchers included 13 questions in the program evaluation form to assess the program. The participants in the experimental group evaluated the program on a five‐point Likert scale. The evaluation form concluded with a part dedicated to gathering opinions and suggestions with open‐ended questions for participants to respond to.

### 2.3 Intervention and Procedure

The online mindfulness‐based streching exercises (MBSE) program included a shortened and revised form of the online MBPE program developed by Terzioğlu et al. (2024) for 8 weeks, 1 day a week for 90 min. The MBPE program consisted of eight sessions, each of which focused on a different topic: understanding mindfulness (session 1), mindfulness and body relationships (session 2), awareness of emotions (session 3), awareness of thoughts (session 4), compassion and self‐compassion concepts (session 5), mindfulness practices in daily life (session 6), aromatherapy (session 7), and conclusion and a new beginning (session 8) (please see Terzioğlu et al. 2024 for detailed content of the program). The MBSE program consisted of six sessions, and the topics of each session were as follows: mindfulness (session 1), awareness of body (session 2), awareness of emotions (session 3), awareness of thought (session 4), compassion and self‐compassion (session 5), and mindful eating and ending the program (session 6). The sixth session of the long form involved mindfulness practices in daily life, and mindful eating exercises were also included in this session. In the sixth session, information about the five senses, mindfulness practices in daily life, mindful eating, and mindful eating were provided. Additionally, aromatherapy session in the original MBPE program was not included in the revised form. The amount and duration of physical exercise sets were revised and reorganized in the online MBSE. Similar to the MBPE, physical exercise was applied in four sessions in the MBSE program.

One session was held each week, and the duration of the sessions was 90 min. Each session included exercises related to mindfulness and self‐compassion. After each group session, participants received homework assignments to practice at home. Participants were invited to integrate these exercises into their daily routines and document them on a Home Practice Form, which was adapted from the Homework Practice Record Form (Segal et al. 2013), and they were required to log assigned exercises and comments on their experiences. Weekly group meetings were consistently conducted on an online platform on the same day and at the same time. Following each exercise during these sessions, the participants were prompted to discuss their thoughts and emotions regarding their experiences with the activity.

After the university's ethics committee granted approval for this study, the students were notified of the web‐based program through their college's email accounts. Seventy‐eight students initially expressed willingness to participate in the study. Students who agreed to participate in the study provided their consent, and were then given scales. Owing to the timing of the sessions and the need for regular attendance at the program, 34 participants declined to participate in the study. Following this, with a random assistant, a total of 44 students were divided into two groups. The first researcher conducted an online pre‐group meeting with the experimental group participants, providing them with more comprehensive details regarding the program's content, duration, and online platform. During the process, there were participants (six participants) who left both groups and a total of 38 people participated in the study.

#### 2.3.1 Online Mindfulness‐Based Streching Exercises Program

As online MBSE was based on the MBPE program (Terzioğlu et al. 2024), it incorporated exercises from the conceptual frameworks of self‐compassion, mindfulness, and acceptance. Exercise is a specific form of planned structured physical activity that aims to improve or maintain fitness (Caspersen et al. 1985). Physical exercise in this study included some stretching movements. The stretching protocol implemented for the study participants includes both static and dynamic stretching exercises, as recommended in the literature, and focuses on general muscle groups (British Medical Association 2013; Kim 2004; Walker 2011). The participants received brief information before each exercise and were guided by the first author during the application. The exercises were performed at the end of the second, third, fourth, and fifth sessions and lasted 30 min. For each movement, two sets and each position in each set were performed within 20–30 s. The rest was given for 10–15 s between the exercises. Transitions were made at a slow pace and accompanied by breathing. Detailed information about the stretching movements performed sequentially is provided in Table 1. In the first session, the content of the program and concept of mindfulness were introduced. Participants performed breathing exercises. The subject of the second session was awareness of the body. The questions “How aware are you of what your body tells you?”, “How is the body affected by the emotional states of the individual?” were discussed. The biological basis of mindfulness was defined, and information was provided regarding the importance of physical exercise. Stretching exercises were performed at the end of the group session. The third session focused on the awareness of emotions and included the importance of physical exercise, stretching exercises, and mindfulness of emotions in the body. The group members were told that whenever they felt a compelling emotion, they could practice mindfulness of the emotion in the body exercise. In this way, it was stated that when there is a challenging emotion in their bodies, it will be released into their minds. Stretching exercises were performed at the end of the group session. The fourth session focused on the awareness of thoughts, and the Pygmalion effect was explained as an example. Nine‐point exercises were performed. This exercise gave an understanding of the tendency to get stuck in a single perspective. Stretching exercises were performed at the end of the group session. The fifth session included compassion and self‐compassion. They were instructed to ask themselves ‘How are you?’ and to answer the question. How to treat I friend? exercise was practiced. This exercise ensured that they realized whether there was a difference between the words that people say to themselves in a difficult time and the words they say to their friends when they are in a difficult time. Loving kindness, meditation, and stretching exercises were performed at the end of the session. They were provided homework to practice loving kindness meditation and stretching exercises. The sixth session began with letting participants go through forgiveness. In this session, mindful eating was discussed. At the end of this session, a 20‐min mindful eating meditation was conducted. After receiving feedback from the participants, post‐test instrumentation was applied.

**TABLE 1:** Stretching exercises.

Sequence	Exercise Name	Set/Time/Repeat
1	Roll‐down stretch	2 set, 20–30 s
2	Back extension stretch	2 set, 20–30 s
3	Seated twist stretch	2 set, 20–30 s, in both directions
4	Clasped hand turns	10 reps, each repetition 5 s
5	Upper back stretch	2 set, 20–30 s
6	Shoulder stretch (external rotator stretches)	2 set, 20–30 s, for each arm
7	Triceps stretch	2 set, 20–30 s, for each arm
8	Seated side stretch	2 set, 20–30 s, in both directions
9	Quadriceps stretch	2 set, 20–30 s, for each leg

### 2.4 Data Analysis

The assumption of normality was verified before the main analysis was conducted. Subsequently, a 2 (group) × 3 (time point) mixed factorial ANOVA was employed to assess the differences between the pre‐, post‐, and follow‐up scores of the experimental and control groups. Due to limited number of available participants, post hoc power analyses were conducted for ANOVA at α = 0.05 (1‐β error probe) using G*Power version 3.1.9.7 (Faul et al. 2007).

## 3 Results

Means and standard deviations for mindfulness, self‐compassion and mental well‐being scores of experimental and control groups are provided in Table 2. The mixed factorial ANOVA results for mindfulness, self‐compassion, and mental well‐being scores of the experimental and control groups are shown in Table 3.

**TABLE 2:** Descriptive statistics for mindfulness, self‐compassion, and mental wellbeing scores by group and time.

Group	Time Point	Mindfulness		Self‐Compassion		Mental Well‐Being	
		*M*	SD	*M*	SD	*M*	SD
Experimental	Pre‐test	61.37	8.91	37.42	8.23	49.74	11.03
	Posttest	68.37	8.85	42.58	7.26	55.32	8.65
	Follow‐up	67.21	10.77	40.58	7.64	54.89	10.24
Control	Pre‐test	64.00	11.68	36.53	7.37	50.11	9.24
	Posttest	58.95	10.46	35.84	8.56	48.58	9.24
	Follow‐up	63.89	5.55	34.58	4.59	49.05	9.62

**TABLE 3:** Mixed factorial ANOVA results for mindfulness, self‐compassion, and mental well‐being scores by group and time.

	Source	*SS*	*df*	*MS*	*F*	*p*	*Partial η²*
Mindfulness	Time (Test)	161.70	2	80.85	1.94	0.151	0.05
	Time × Group	690.05	2	345.03	8.30	0.001*	0.19
	Error (Time)	2994.91	72	41.60			
Self‐Compassion	Test	101.73	1.92	52.90	2.16	0.125	0.06
	Time × Group	192.33	2	96.16	4.08	0.021*	0.10
	Error (Time)	1695.26	72	23.54			
Mental Well‐Being	Time (Test)	105.38	2	52.69	1.71	0.187	0.05
	Time × Group	284.54	2	142.27	4.64	0.013*	0.11
	Error (Time)	2208.73	72	30.67			

* *p* < 0.05.

As indicated in Table 3, there was a significant time × group interaction, F_(2, 72)_ = 8.30, *p* = 0.001, partial η² = 0.19, indicating that the change in mindfulness scores over time differed between groups. Post hoc pairwise comparisons for mindfulness scores revealed significant increases in the experimental group from pre‐test to posttest (*p* = 0.005), while no significant differences were found between the posttest and follow‐up test (*p* = 1.00) (Table 4). For self‐compassion scores, there was a significant time × group interaction, F_(2, 72)_ = 4.08, *p* = 0.021, partial η² = 0.10, indicating that the change in self‐compassion scores over time differed between groups (Table 3). Post hoc pairwise comparisons for self‐compassion scores revealed significant increases in the experimental group from pre‐to post‐test (*p* = 0.010), whereas no significant differences were found between the post‐test and follow‐up test (*p* = 0.493) (Table 4).

**TABLE 4:** Pairwise comparisons of mindfulness, self‐compassion and mental well‐being scores across time points by group.

Group	Comparison	Mindfulness		Self‐Compassion		Mental Well‐Being	
		*Mean Diff*.	*p*	*Mean Diff*.	*p*	*Mean Diff*.	*p*
Experimental	Pre versus Post	−7.00	0.005*	−5.15	0.010*	−5.57*	0.017*
Experimental	Post versus Follow‐up	1.16	1.00	2.00	0.49	0.42	1.00
Control	Pre versus Post	5.05	0.054	0.68	1.00	1.52	1.00
Control	Post versus Follow‐up	−4.95	0.066	1.26	1.00	‐0.47	1.00

* *p* < 0.05.

For mental well‐being scores, there was a significant time × group interaction, F_(2,72)_ = 4.64, *p* = 0.013, *p* = 0.013, and partial η² = 0.11, indicating that changes in mental well‐being scores over time differed between the experimental and control groups (Table 3). Post hoc comparisons within the experimental group showed a significant increase in mental well‐being from pre‐to post‐test (*p* = 0.017), while no significant differences were found between the mental well‐being post‐test and follow‐up scores (*p* = 1.00). The control group's mindfulness, self‐compassion, and mental well‐being scores showed no significant differences at any time point.

Upon completion of the study, a satisfaction survey was administered to students who participated in the psycho‐educational program. Table 5 presents the means and standard deviations of the participants' evaluations of the program.

**TABLE 5:** Means and standard deviations for the evaluations (*n* = 19).

	*Mean*	*sd*
I was willing to attend the group.	4.95	0.23
Sessions were regularly held.	4.95	0.23
We communicated effectively with the group leader.	4.95	0.23
Group members effectively communicated with each other.	4.68	0.48
Group exersices were adequate throughout the group process.	4.84	0.50
The activities carried out during the sessions attracted my attention.	4.89	0.32
The content of group sessions were adequate.	4.84	0.50
Individual differences were respected throughout the process of group sessions.	5.00	0.00
During the sessions, I was able to communicate my feelings and ideas in a genuine and forthright manner.	4.68	0.58
The execises in the program had a positive impact on me.	4.95	0.23
I reached to my goals that I set throughout the group process.	4.53	0.61
I can recommend this to my friends.	4.95	0.23
The program was effectively implemented by the leader.	4.89	0.46

As shown in Table 5, there was 97% satisfaction with the program. In the participants' comments section of the program evaluation form, sample comments were as follows: *“I think it is an experince that every person who wants to know and accept themselves should participate in, I was very happy to be with a very fun and interested group throughout the process,” “The program was a highly enjoyable, structured, and informative experience. I gained awareness of the issues that I was unaware of. I became more conscious and knowledgeable about the issues that I was aware of,” “It was a program that taught me to stay in the moment. I willingly participated in every session, and I think that everyone caught a place from their own lives in most of the sessions.”*

## 4 Discussion

Our study investigated the effects of a brief online program integrating mindfulness and stretching exercises on health sciences students across three domains: mindfulness, self‐compassion, and mental well‐being. The results indicated that following the six‐session online MBSE, there was a significant difference in the change in mindfulness scores between the experimental and control groups over time. Specifically, MBSE led to a significant increase in mindfulness among students in the experimental group, whereas no significant difference was observed in the control group. Notably, the positive effects of the program were maintained for 3 months in the experimental group. The observed increase in mindfulness in the experimental group aligns with the findings of previous studies. For example, Gonzalez‐Hernandez et al. (2022) EEG data showed that a mindfulness program reduced students' anxiety levels during exams. Similarly, Hearn and Stocker (2022) found that exams elevated cortisol levels. However, following a mindfulness intervention conducted 7 days before the exams, medical students with higher mindfulness scores exhibited decreased salivary cortisol and perceived stress levels, which was also associated with improved exam performance. Additional research supports these findings. Song and Lindquist (2015) reported that nursing students who participated in an 8‐week mindfulness‐based stress reduction (MBSR) program, consisting of 2‐h weekly sessions, experienced significant reductions in depression, anxiety, and stress, along with increased mindfulness. Likewise, Collard et al. (2008) found that students involved in a mindfulness‐based cognitive therapy (MBCT) program demonstrated significant improvements in mindfulness and reductions in negative affect. Furthermore, a 6‐week mindfulness‐based intervention by Chiodelli et al. (2018) also led to enhanced mindfulness levels among students.

The results of this study demonstrated that, following participation in the online MBSE program, health sciences students in the experimental group experienced a statistically significant increase in self‐compassion. This positive effect persisted for a duration of 3 months. These findings are supported by previous studies. For instance, university students who participated in a mindfulness‐based stress reduction (MBSR) program showed greater increases in mindful self‐compassion than those in a control group (MacDonald and Neville 2023). Similarly, both mindfulness‐based mobile applications and in‐person mindfulness training programs have been shown to enhance self‐compassion and mindfulness levels among healthcare students (Orosa‐Duarte et al. 2021). In another study, clinical and health psychology trainees who participated in MBSR and mindful self‐compassion (MSC) programs exhibited significant improvements. Specifically, those in the MBSR group showed reductions in anxiety and depression, while participants in the MSC group demonstrated notable increases in both mindfulness and self‐compassion compared to the control group (Jiménez‐Gómez et al. 2022). Further evidence from Evans et al. (2018) indicates that participants in an MBSR program experienced significant improvements in mindfulness and self‐compassion. Importantly, this study also found that self‐compassion mediates the relationship between mindfulness and well‐being following MBSR training.

Internet‐delivered mindfulness and relaxation programs have proven effective for both healthy and highly distressed students, resulting in clinically significant reductions in stress levels (Messer et al. 2015). During exam periods, students who received mindfulness training reported lower levels of psychological distress and were less likely to meet clinical distress criteria (Galante et al. 2018). Cavanagh et al. (2013) also reported that a short‐term, individualized online mindfulness intervention led to significant improvements in mindfulness and reductions in stress, anxiety, and depression among university students compared to a control group. Moreover, a brief three‐session mindfulness group intervention was found to increase students' mindfulness levels, while reducing symptoms of depression, anxiety, and stress (Parcover et al. 2018).

According to our results, group participation increased health sciences students' mental well‐being levels, and this effect was maintained for 3 months. Previous research, has shown that mindfulness‐based programs can effectively decrease perceived stress, and enhance mental well‐being (Fazia et al. 2023). Similarly, engaging in meditation has been demonstrated to enhance mindfulness and mental well‐being, while concurrently reducing stress levels (Zollars et al. 2019). Short‐term, low‐ to moderate‐intensity aerobic exercise was also found to be effective in reducing depression and perceived stress (Herbert et al. 2020). Similarly, moderate‐intensity aerobic exercise reduced tension/anxiety, depression/dejection, and confusion after exercise. In the same study, it was observed that it is not always a more intense exercise, but that an appropriate dose of exercise is more effective in supporting mental health (Moses et al. 1989). Our results are consistent with those of the previous studies.

In a study conducted in Switzerland during the first wave of the COVID‐19 pandemic, mindfulness practice enhanced practitioners' resilience levels, indicating that meditation helps manage challenges during traumatic situations. Furthermore, participants engaging in physical activity maintained consistent resilience scores and reduced depression scores over time (Antonini Philippe et al. 2021). In a study conducted in Hong Kong, a positive relationship was found between physical activity, self‐compassion, and mental well‐being within the scope of the EXSEM‐SC model, and physical activity was found to be effective in protecting adolescents' psychological resilience during the pandemic (Wong et al. 2023). Physical activity has been found to increase students' psychological well‐being (Rehor, et al. 2001). Similarly, individuals who exercise at least two–three times a week have been shown to experience less depression, anger, cynical distrust, and stress. A consistent relationship has been found between regular physical activity and improved psychological well‐being (Hassmen et al. 2000). These findings underscore the protective and promotive effects of physical exercise on both physical and psychological health.

In summary, these findings suggest that interventions based on physical activity, integrated with mindfulness, hold strategic significance for university students, contributing not only to physical fitness, but also to general well‐being. Our findings provide additional support for the effectiveness of mindfulness‐based physical activity in enhancing students' well‐being in health‐related fields. The distress that university students experience in their social and academic lives or in processes related to themselves may affect academic performance and self‐compassion levels. Our findings suggest that online MBSE is effective in enhancing mindfulness, self‐compassion, and mental well‐being among health sciences students.

### 4.1 Limitations and Recommendations

Several constraints must be considered in this study. The primary limitation is the restricted sample size. This necessitates a cautious interpretation of the findings. Nevertheless, the power analysis demonstrated reliable results, with values of 0.96 for mindfulness, 0.88 for self‐compassion, and 0.80 for mental well‐being in post‐test comparisons at a significance level of α = 0.05. Although these outcomes are promising, it is recommended to evaluate the program's effectiveness using larger participant groups to obtain more reliable power analysis results (Perugini et al. 2018). This study focused on students from specific health‐related programs, including dentistry, midwifery, nutrition and dietetics, physiotherapy, and rehabilitation. As a result, these findings cannot be broadly applied to students in other healthcare disciplines. Furthermore, we collected data only through self‐report scales. The data collection instruments employed in this study demonstrated high validity and reliability; however, they were constrained by the specific characteristics they measured. It is assumed that the university students participating in the study answered the measurement tools given to them sincerely and objectively. However, in this case, the responses may reflect biases arising from the students' expectancy effects. In future studies, methods that measure physiological changes should be used. Next, the experimental group results might stem from uncontrolled confounding variables, leading to a potential misinterpretation of the independent variable's effect on the dependent variable and diminishing result validity. Future studies should incorporate a placebo control group to manage these variables better. In addition, in future studies, short‐ and long‐form mindfulness‐based interventions can be applied together and compared. To evaluate their influence, researchers can measure factors such as the number and length of sessions, frequency of daily practice, and variety of mindfulness exercises. Additionally, factors such as intervention length and the effects of online versus face‐to‐face implementation can be evaluated to determine their influence on program effectiveness. Despite these limitations, our findings have several implications. The study indicates that the mindfulness‐based stretching exercises program significantly enhances self‐compassion, mindfulness, and mental well‐being, and contributes significantly to web‐based mindfulness interventions for university students. Our results suggest that, despite considerable distress faced by health sciences students during their academic journey, mindfulness‐based exercise may bolster their well‐being. Online mindfulness training is a cost‐effective intervention that can be delivered to all students or groups with special needs to improve well‐being. Thus, university counseling centers should incorporate online mindfulness‐based exercise programs in their services.

## Ethics Statement

The authors confirm that this study was conducted in accordance with the 1964 Declaration of Helsinki and its later amendments or comparable ethical standards. This study was approved by the Ethics Committee of Akdeniz University (No. 31/10/2023‐450).

## Consent

Written informed consent was obtained from all participants. The manuscript does not contain any clinical studies or patient data.

## Data Availability

The data that support the findings of this study are available on request from the corresponding author. The data are not publicly available due to privacy or ethical restrictions. Data will be made available on request.
